# Supplementary material for: The interactome of intact mitochondria by cross-linking mass spectrometry provides evidence for coexisting respiratory supercomplexes
Source: Mol Cell Proteomics. 2017 Dec 8;17(2):216–32. doi: 10.1074/mcp.RA117.000470 (PMC5795388; doi:10.1074/mcp.RA117.000470)
Supplement: Supplemental Data [file supp_RA117.000470_133922_0_supp_23978_fzffwf.zip › spectra_annotation/mito_DR_spectra_annotation/78-1-6-1-26-1.pdf]

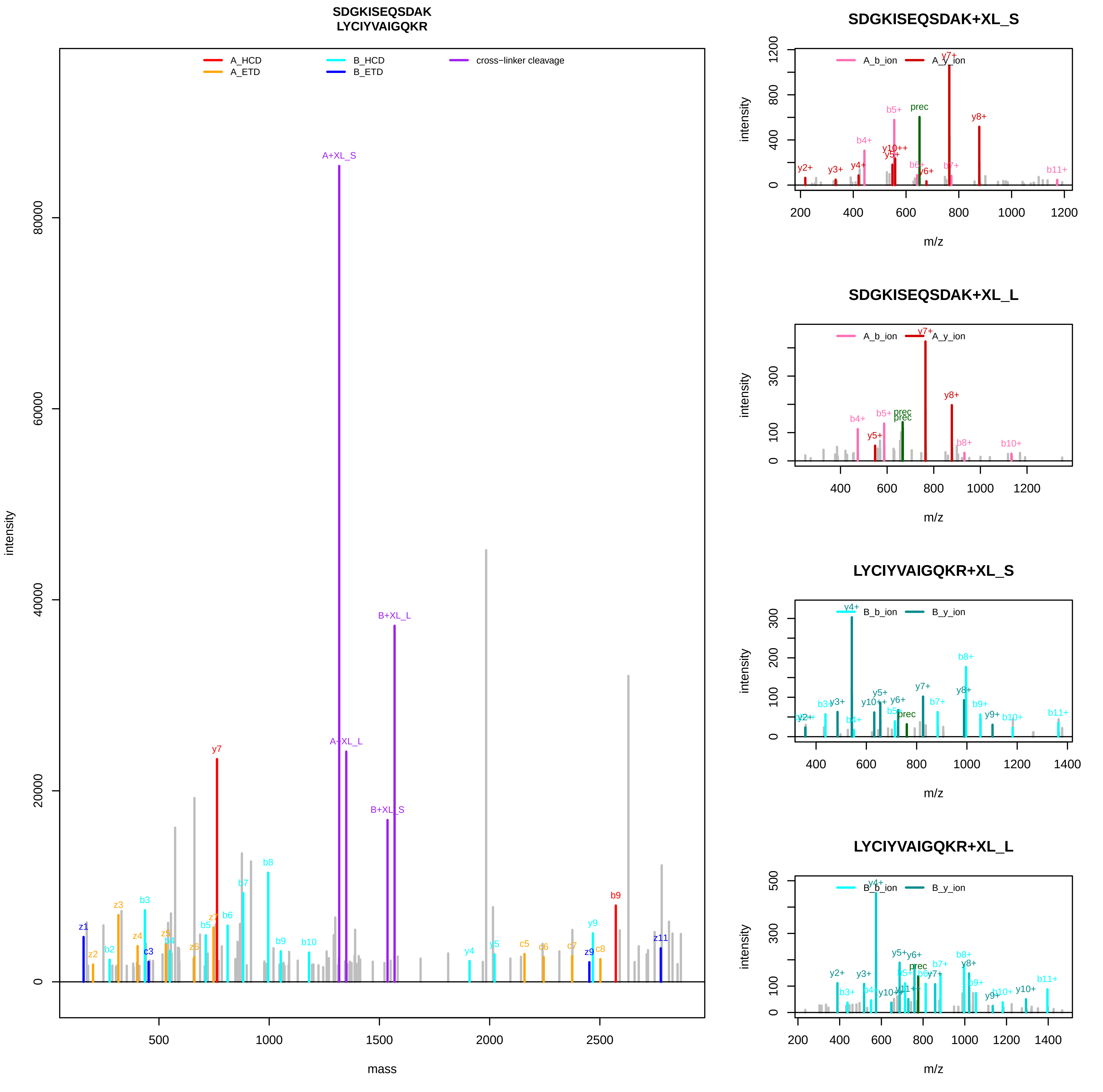
SDGKISEQSDAK
LYCIYVAIGQKR
A_HCD
A_ETD
B_HCD
B_ETD
cross−linker cleavage
A+XL_S
B+XL_L
A+XL_L
B+XL_S
intensity
mass
0
20000
40000
60000
80000
500
1000
1500
2000
2500
z1
z2
b2
z3
b3
z4
c3
z5
b4
z6
b5
z7
b6
y7
b7
b8
b9
b10
y4
y5
c5
c6
c7
z9
y9
c8
b9
z11
SDGKISEQSDAK+XL_S
A_b_ion
A_y_ion
y2+
y3+
y4+
b4+
y5+
y10++
b5+
prec
b6+
y6+
y7+
b7+
y8+
b11+
m/z
SDGKISEQSDAK+XL_L
b8+
b10+
LYCIYVAIGQKR+XL_S
B_b_ion
B_y_ion
y3+
y6+
y8+
b9+
y9+
y10+
LYCIYVAIGQKR+XL_L
b3+
b6+
y11+
